# The Influence of Position upon Pain

**Published:** 1909-01-23

**Authors:** 


### THE INFLUENCE OF POSITION UPON PAIN.

In discussing the pain associated with the various organs it is often desirable to emphasise its dependence on definite positions of the body such as the dorsal, the lateral, and so forth, which frequently appear to bear a distinct relation to the sensation (Schmidt*). Observations of this sort lead to the characterisation of certain "positions of maximum pain," which term may be applied to those positions which give rise to a pain which previously did not exist or which increase the intensity of a pain already present. In so far as the painful position depends on tenderness to pressure of superficial structures, as in joint affections, etc., it has little diagnostic interest. In gastric ulcer the existence of a painful position has been accorded a somewhat unjustifiable degree of importance from the standpoint of differential diagnosis, and for this reason the interpretation of the symptom is not always clear cut.

As a matter of fact, painful positions may be discovered in connection with the pain complexes of the most varied organs, and this therefore points to uniformity in the mechanism of their origin. For example, there occur "painful positions" in diseases of the gall bladder, of the vermiform appendix, in abdominal tumours, aneurysm of the aorta, pericarditis and so forth. Rudolph Schmidt has noted that even in intracranial processes, such as cerebellar tumours, there may be painful positions in regard to the headache, which occurs on the side opposite to that of the hemisphere in which the tumour is situated, and may depend on the pressure of the growth on the vena magna galeni or the aqueduct of Sylvius. In the majority of cases the most general cause of pain is to be sought for in a change of position of the diseased organ, such as occurs in certain positions of the body.

Painful traction on diseased organs is likely to result—especially in cases of inflammatory processes in the immediate neighbourhood of the structures involved, as in perigastritis, appendicitis, periaortitis, etc.—in those positions of the body in which the organ is deprived of its firm support. This is ordinarily the case in the position on the side opposed to the lesion, and the resulting pain will depend on the degree of sensibility caused by the inflammation and on the intensity of the traction (*i.e.* on the weight and mobility of the displaced mass). Of course other factors also come into play, such as pressure on neighbouring nerve trunks, as in aneurysms, tumours, and the like, as well as secondary pressure effects on muscular hollow organs like the stomach, intestine, or ureter. A special mechanism depending on the local peculiarities of the tissues involved underlies the position of maximum pain in certain diseases of the aorta or the coronary arteries. It is well known that the horizontal position may sometimes provoke attacks of angina pectoris.

What light is thrown on the problem of differential diagnosis by the discovery that there is in a given case a position of maximum pain? If the problem presenting itself for decision is whether the pain is organic or functional in nature, the existence of a painful position is in favour of an organic lesion. Thus in cases of mediastinal new growth, including carcinoma of the œsophagus, aneurysm of the thoracic and abdominal aorta, gastric ulcer, etc., the nature of the attendant pain is not rarely misunderstood and is considered as being a functional manifestation of a neurosis. Under these conditions the demonstration that there is a distinct position of increased pain may be of decisive moment.

The presence of a painful position always indicates the advisability of a search for the organ or new growth causing it, and the location of the sensation attending the painful position will correspond to the situation of the organ or new growth in question. The detection of deeply situated tumours involving, for example, the pancreas or œsophagus, is often a matter of difficulty, and in these cases the presence of a painful position may be taken as being corroborative of doubtful palpatory evidence. The occurrence of a painful position points towards a localised process, especially in dealing with the abdomen, even when the pain appears to be diffuse as in some cases of appendicitis, intestinal cancer, cholelithiasis, nephrolithiasis, etc., and so may be of service in differentiating an ordinary intestinal colic from similar painful sensations originating in appendicular disease or localised carcinoma.

The lateral posture is a painful position *par excellence*, for it involves the most favourable conditions for abnormal displacement and traction. The dorsal position—for instance in retroperitoneal lesions—or the sitting posture may also come into question however. In the latter case the symptom is usually difficult to interpret. Pain in the small of the back and in the flanks, after long sitting, especially if the body is inclined forward, may be caused by swollen abdominal organs. These pains do not, however, appear very promptly, but only after long continuance of the position, and the pain may sometimes also be attributed to fatigue of the dorsal musculature.

* Schmidt, Rudolph: "Pain." See Review in The Hospital, page 201.

